# A Study on the Thermal Effect by Multi Heat Sources and Machining Characteristics of Laser and Induction Assisted Milling

**DOI:** 10.3390/ma12071032

**Published:** 2019-03-28

**Authors:** Jae-Hyeon Ha, Choon-Man Lee

**Affiliations:** School of Mechanical Engineering, Changwon National University, 20, Changwondaehak-ro, Uichang-gu, Changwon-si, Gyeongsangnam-do 51140, Korea; rednadayg@hanmail.net

**Keywords:** Laser-Induction assisted milling, Multi heat sources, Thermal effect, Electromagnetic-thermal coupled analysis, Machining characteristics

## Abstract

Thermally assisted machining (TAM) is an effective method for difficult-to-cut materials, and works by locally preheating the workpiece using various heat sources, such as laser, induction, and plasma. Recently, many researchers have studied TAM because of its low manufacturing costs, high productivity, and quality of materials. Laser assisted machining (LAM) has been studied by many researchers, but studies on TAM using induction or plasma heat sources, which are much cheaper than lasers, have been carried out by only a few researchers. Lasers have an excellent preheating effect, but are expensive, and the temperature of the heated workpiece drops quickly. Here, multi heat sources were used to solve the shortage in supplied heat source with a single heat source. Induction was applied as an additional heat source. The purpose of this study is to analyze the thermal effect and temperature distribution of single heat source and multi heat sources, and compare the machining characteristics according to heat source types. In order to analyze the preheating effect according to the feed rate of the heat sources, a temperature measurement experiment using thermocouples was carried out, and the efficiency of the thermal effect using multi heat sources was verified. In addition, the effectiveness of the thermal analysis results was verified by comparison with the measured temperature distribution. The machining characteristics of Inconel 718 and Ti-6Al-4V with laser, induction, and laser-induction assisted milling (LIAMill) were analyzed, by cutting force and surface roughness.

## 1. Introduction

Recently, interest in thermally assisted machining has been increasing worldwide as a method to process difficult-to-cut materials at low cost and high efficiency. TAM is a method of locally preheating the material using various heat sources, such as laser, induction, and plasma, ahead of the cutting tool. In particular, as laser technology has improved, many researchers have studied LAM, which effectively preheats the difficult-to-cut materials using a laser beam with high density energy [1,2]. While the demand for difficult-to-cut materials is increasing in various fields, such as automobile and aerospace industries, they remain difficult to process because of their high hardness and brittleness. Jonathan and Shin [3] studied laser assisted micro-milling (LAMM), which can be used in the micro electro mechanical systems (MEMS) industry. LAMM reduced the large edge burrs in machining of Inconel 718 and Ti-6Al-4V more than conventional machining. Xiao et al. [4] studied laser additive manufacturing using a quasi-continuous wave laser to control the laves phase of Inconel 718 alloy, which has been widely used in nuclear reactors, gas turbines, and aero-engines. Guimaraes and Jonas [5] studied the recrystallization and aging effects of Waspaloy and Inconel 718. The cylindrical shaped workpieces of Waspaloy and Inconel 718 were hot compressed. Dynamic recrystallization temperature and stress-temperature curve was determined.

Laser assisted turning (LAT) is relatively simple to control and develop compared to laser assisted milling (LAMill), and has already been studied and commercialized in many countries. Many researchers have investigated the efficiency of LAT for various materials and machining conditions. Ayed et al. [6] studied LAM of Ti-6Al-4V alloy. The study showed that the Ti-6Al-4V alloy has high mechanical characteristics and is biologically compatible with the human body. Lee et al. [7] studied LAM of silicon nitride ceramics. Ceramics are widely used in many industries, such as engines, medical applications, aerospace, and ocean fields. Venkatesan et al. [8] studied LAM of Inconel 718. The study showed that the laser power, approach angle, cutting speed, and feed rate were considered as a parameter of modeling in LAM of Inconel 718. Optimal condition was determined via parametric modeling. Razavi et al. [9] studied LAM of Inconel 718 using a pulsed laser with 80 Hz frequency. The study showed that when the workpiece temperature was about 540 °C, the machining specific energy reduced by 35%, and surface and tool wear were also reduced by 22% and 23%.

On the other hand, LAMill has been actively studied because it has proven to be difficult to develop. The reason for this difficulty is that the size and shape of the laser heat source, when used to preheat complex shaped workpieces, constantly change. Also, it is difficult to control how the laser heat source proceeds along various tool paths.

Bermingham et al. [10] studied laser assisted milling strategies with different cutting tool paths and verified their effectiveness with the computer aided manufacturing (CAM) program. Lee et al. studied the cutting forces and surface roughness of the workpiece when processing three-dimensional shapes, such as ellipses and cylinders, using various machining methods [11,12]. Wiedenmann et al. proposed a new machining strategy using laser assisted milling and verified the effectiveness by measuring cutting force and material removal rate [13]. Wang et al. studied the coated tool wear characteristics of nickel alloys using laser assisted milling [14]. Kim et al. proposed a prediction equation for the cutting force and preheating temperature of Inconel 718 and AISI 1045 steel using laser assisted milling [15]. Lee et al. conducted machining tests using eco-friendly machining methods, such as MQL, cryogenic, and LAM for titanium alloys. They compared the tool wear, cutting force, and power consumption for each machining method using experimental results [16]. Shin et al. used thermal and mechanical modeling analysis to investigate the machining characteristics of difficult-to-cut materials using laser assisted side cutting [17]. 

As noted above, studies on LAMill are still being performed. The latest LAMill research trends involve the selection of various tool paths ahead of the laser, establishing machining strategies for processing various three-dimensional shapes, and to increase tool life. Lee et al. [18] investigated the trend of laser assisted milling devices. Laser assisted milling was classified as laser assisted face milling, laser assisted end milling, laser assisted turn-mill, and laser assisted ball end milling. They proposed the future research directions as the moving control system of the laser module and development of a cutting tool with high-heat resistance and high-hardness. Bermingham et al. [19] studied tool life and wear mechanisms in laser assisted milling of Ti-6Al-4V. The tool life was investigated in various machining conditions, such as laser assisted milling, dry machining, milling with flood emulsion, milling with minimum quantity lubrication (MQL), and a hybrid laser and MQL approach. Lee et al. [20] performed a project of "A new conceptual 3-dimensional Laser Assisted Machining System". They studied laser transfer module and 3D shape machining via laser assisted milling.

However, because the workpiece with LAM is only preheated by a laser heat source, a problem developed, in that the supplied heat source was insufficient. In order to solve this problem, the present author has studied various preheating paths to prevent temperature drop [21,22]. In order to prevent the temperature drop in the workpiece, a preheating path using a laser with zig-zag and back-and-forth methods is proposed. The preheating effect was increased, but there was a disadvantage when the processing time was increased, because it takes time to adequately preheat the workpiece before machining, and this makes it difficult to apply to a long machining path. Therefore, in this study, a three-dimensional hybrid machining system using multi heat sources was developed, and the defects of LAM due to single heat source were resolved. The additional heat sources selected for the system can be cheaper than lasers, such as induction or plasma heat sources. Research on TAM using induction or plasma heat sources are being carried out by just a few researchers [23,24]. Kim and Lee [25] studied optimal machining parameters of the induction assisted machining. Machinability of coated and uncoated cutting tools were evaluated using the Taguchi method in induction assisted machining. López de Lacalle et al. [26] studied Plasma Assisted Machining (PAM) of heat resistant alloys, such as nickel-based alloys (Inconel 718) and cobalt-based alloys (Haynes 25). The cutting force decreased by 30% compared with the conventional milling process, derived from the numerical and experimental analyses.

**Figure 1:**
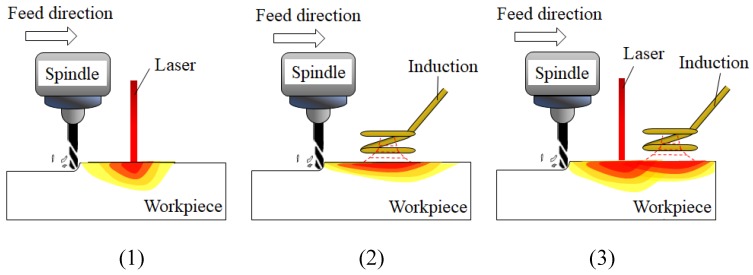
The schematic diagram of thermally assisted machining with (**1**) Laser assisted milling, (**2**) Induction assisted milling, and (**3**) Laser-induction assisted milling.

However, there has been no research on the preheating and machining of a workpiece using multi heat sources anywhere in the world. Using multi heat sources prevents the preheating temperature of the workpiece from dropping as rapidly and the machining speed is increased by maintaining the preheating temperature. Figure 1 shows a schematic diagram of TAM using laser and induction heat sources.

The purpose of this study is to measure the thermal effect of the workpiece using laser, induction, and laser-induction heat sources, and to verify the efficiency of the preheating effect produced by multi heat sources. A thermal analysis was performed using the finite element method (FEM), and based on the results, the proper preheating temperature and effective depth of cut of Inconel 718 and Ti-6Al-4V were selected. Also, the effectiveness of the thermal analysis results were verified by comparing the measured maximum temperature. Then, TAM was performed to analyze the machining characteristics, such as the cutting forces and surface roughness of each heat source. The significance of multi heat sources was verified by comparing the machining characteristics of each preheating method.

## 2. Thermal Analysis

TAM is a method of efficiently machining difficult-to-cut materials by softening the workpiece using a heat source before machining with a tool. Before conducting a machining experiment it is necessary to first determine the proper preheating temperature, because the mechanical strength of a specific material depends on the temperature. If the preheating range of the material is not appropriate, changes in the material properties and phases may occur, and the machining efficiency may be decreased during TAM [5,27]. In this study, the thermal analysis was performed to determine the proper preheating temperature and effective depth-of-cut (DOC) for the experimental materials [12,15]. DOC means depth of cut. In this study, the depth of cut for TAM considering the heat affected zone that was obtained through the thermal analysis was determined. The electromagnetic-thermal coupled analysis of the laser-induction heat source was performed using the ANSYS workbench program (ANSYS, Canonsburg, PA, USA), and the electromagnetic analysis of induction heat source was performed with the Maxwell program.

### 2.1. Analysis Conditions

Before performing the electromagnetic-thermal coupled analysis, an electromagnetic analysis of the induction heat source using the Maxwell program was performed. When preheating using an induction heat source, the heat energy is transferred to the workpiece via eddy currents. As a high frequency alternating current flows through the coil, a lot of eddy currents are induced in the workpiece. The induction heating analysis model consists of a coil for electromagnetic induction, a workpiece, and air space. The diameter of the induction coil was 10 mm. As a boundary condition for the electromagnetic analysis, a high frequency current of 300 kHz was applied, and the current flow was applied to a section of the heating coil. 

The electromagnetic analysis can be described using Maxwell equations. The Maxwell equations for heat transfer are as follows in Equations (1)–(4).

(1) $$\nabla \times H=J+\frac{\partial D}{\partial t}$$

(2) $$\nabla \times E=-\frac{\partial B}{\partial t}$$

(3) $$\nabla \cdot D=\rho_{c}$$

(4) ∇·B=0

The thermal analysis can be described using governing equations. The governing equations of the three-dimensional heat transfer are as follows in Equation (5).

(5) $$\frac{\partial T}{\partial t}=\frac{k}{\rho_{t}C}\left(\frac{\partial^{2}T}{\partial x^{2}}+\frac{\partial^{2}T}{\partial y^{2}}+\frac{\partial^{2}T}{\partial z^{2}}\right)+Q$$

where *H* is the magnetic field intensity or B/μ, *J* is the conduction current density or σE, *D* is the electric displacement or εE, t is time, *E* is the electric field intensity, *B* is the magnetic flux density, and $\rho_{c}$ is the charge density, and where $k\;,\;\rho_{t},\;C,\;T,\;t$, and Q are, respectively, thermal conductivity, density, specific heat, temperature, time, and heat generation rate.

**Figure 2:**
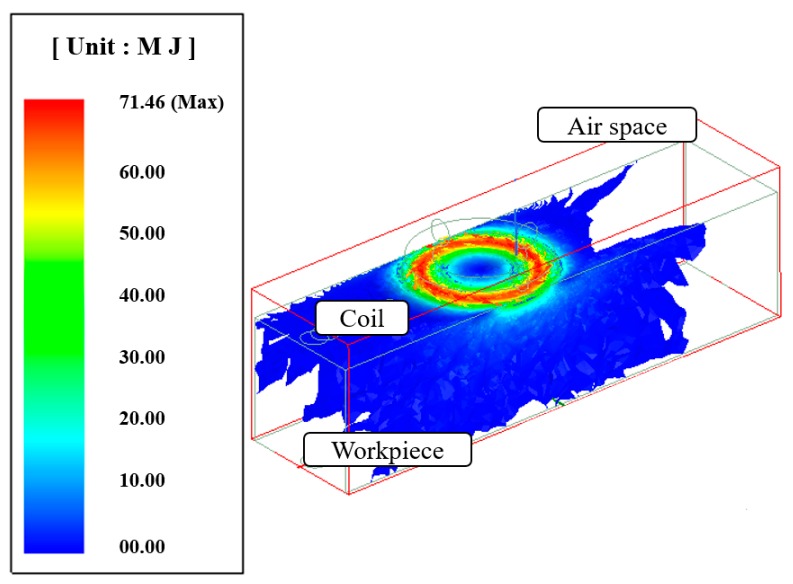
The current distribution in the workpiece.

**Figure 3:**
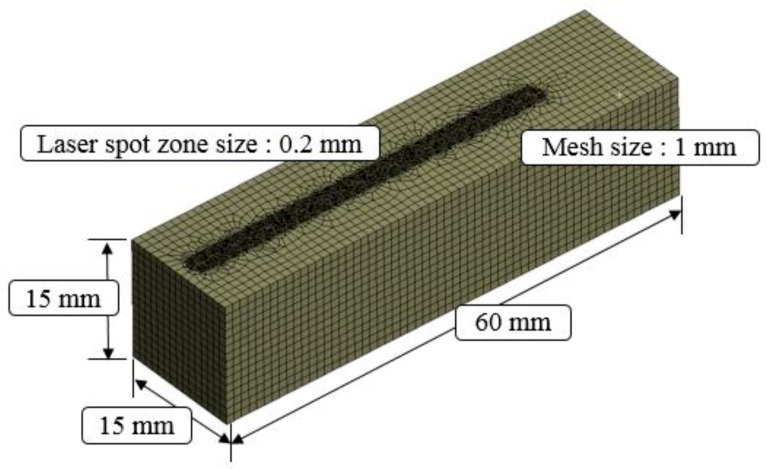
Thermal analysis model.

Figure 2 shows the current distribution in the workpiece. The analysis results of the induction heat source were transferred to the ANSYS workbench program and an electromagnetic-thermal coupled analysis was performed. Mesh generation and boundary conditions were set to perform the analysis of the laser-induction heat sources. The hex dominant method was applied to the mesh generation for thermal analysis. The mesh size was divided by 0.2 mm on the upper surface in the laser spot zone and 1 mm on the rest of the workpiece except for the laser spot zone. The number of elements and nodes in the analysis model was 69,681 and 203,045, respectively. The distance between the laser and induction heat source was set at 3 mm. Figure 3 shows the analysis model for the laser-induction electromagnetic-thermal coupled analysis. The specific heat and thermal conductivity of the two materials were obtained from previous studies, as shown as Table 1 and Table 2 [15,28], because the thermal analysis using a single heat source from laser and induction was performed in previous studies [29,30]. Heat flow is given sequentially to heat source geometry according to the feed rate by the overlap along the tool path.

In the thermal analysis using the laser heat source, the emissivity and absorptivity that depend on the surface roughness and material characteristics should be considered in the analysis and experiments that use the laser power control method [15,31]. Laser power control method is a method of selecting a laser power and maintaining constant power. When laser power control method is used, absorptivity of the workpiece to laser irradiation is very important because the heat flow input to the material depends on the absorptivity. Therefore, absorptivity is determined by quantum photodetector, integrating sphere, or experimental method using the following Equation (6).

(6) $$A=\frac{m\cdot C_{p}\cdot \Delta T}{E_{I}\cdot \Delta t}$$

where m, $C_{p}$, ΔT, $E_{I}$, and Δt are the weight of the workpiece, specific heat of the workpiece, temperature rise of the workpiece, laser power, and preheating time of the diode laser, respectively [15]. In this study, we have used the temperature control method, in which the desired preheating temperature is maintained by automatically controlling the laser power using the pyrometer. Therefore, the amount of heat flux that satisfies the preheating temperature of the set surface is applied to the numerical analysis model. the heat flux to keep the surface temperature at 600 °C (Ti-6Al-4V) and 900 °C (Inconel 718) regardless of absorptivity was set and input to the thermal analysis model. In this chapter, only one thermal analysis of the laser-induction heat sources was performed in order to understand the temperature distribution generated by multi heat sources.

### 2.2. Thermal Analysis Results

#### 2.2.1. Inconel 718

For difficult-to-cut materials such as Inconel 718, the mechanical strength changes depending on the preheating temperature irradiated to the material [32]. The tensile strength of Inconel 718 is reduced sharply in the range of approximately 700 to 900 °C. The recrystallization temperature of Inconel 718 occurs at over 950 °C and melting temperature is approximately 1260 to 1336 °C. Therefore, to avoid changing the crystal grain during the preheating and machining of Inconel 718, the proper preheating temperature of Inconel 718 was set to 900 °C in this study [4,11]. 

**Figure 4:**
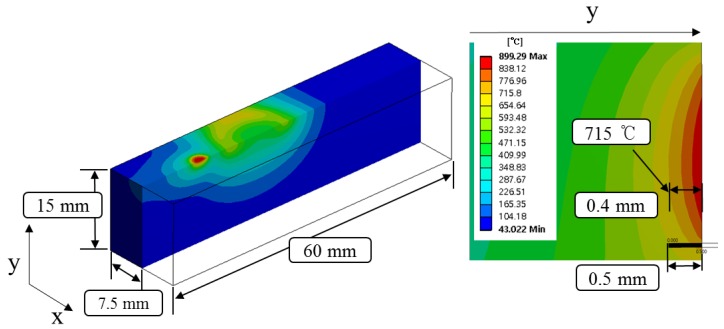
The analysis results for Inconel 718.

Figure 4 shows the temperature distribution and effective depth of cut of Inconel 718 by multi heat sources using laser and induction. Based on the thermal analysis results, the effective depth of cut was selected to be 0.4 mm, which is around 715 °C in the set temperature range.

#### 2.2.2. Ti-6Al-4V

The tensile strength of Ti-6Al-4V drops sharply in the range of approximately 450 to 600 °C. A phase change in Ti-6Al-4V can occur at over 800 °C. In particular, the transformed β (Widmanstatten) occurs at approximately 970 [33]. Surface oxidation occurs at approximately 1100 °C. Therefore, to avoid changing the crystal grain during the preheating and machining of Ti-6Al-4V, the proper preheating temperature of Ti-6Al-4V was set to 600 °C in this study [27,28].

**Figure 5:**
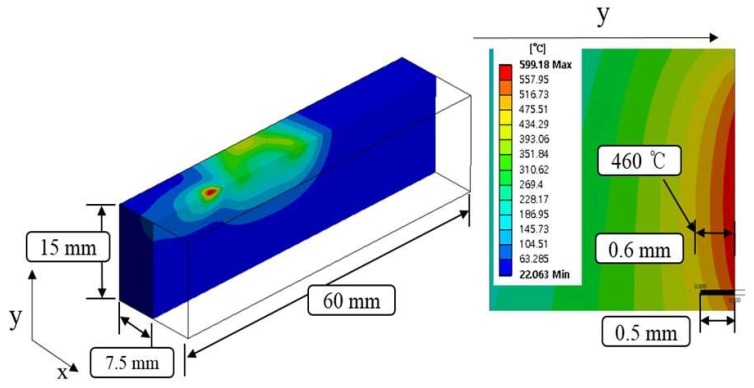
The analysis results for Ti-6Al-4V.

Figure 5 shows the temperature distribution and effective depth of cut of Ti-6Al-4V by multi heat sources using laser and induction. In consideration of the thermal analysis results, the effective depth of cut was selected to be 0.6 mm, which is around 460 °C in the set temperature range.

## 3. Thermal Measurement Experiment

### 3.1. Experimental Set-Up

The thermal measurement experiments to determine the temperature distribution of the laser, induction, and laser-induction heat sources was performed using thermocouples. The preheating temperature set for the device was checked to ensure it was properly applied to the workpiece, and the temperature of the workpiece was checked to ensure it was maintained over time after the heat source passed. 

**Figure 6:**
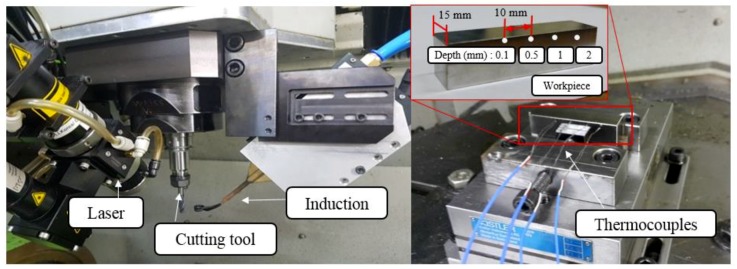
Experimental set-up and workpiece for temperature measurement.

Figure 6 shows the experimental set-up used in this study, and Table 3 lists the equipment used in the experiment. The thermocouples used were a K-type, which can measure at high temperatures, and the temperature measurement positions of the workpiece were located at four holes. The diameter of the holes was 1.6 mm and the depths for temperature measurements were 0.1 mm, 0.5 mm, 1 mm, and 2 mm, respectively. The distance between each thermocouple was 10 mm [34,35]. The temperature data measured using the thermocouples was recorded by a data logger. The temperature distribution was measured separately for the laser, induction, and laser-induction heat sources according to the feed rate of the heat sources. Hwang et al. [22] proposed 3 types of preheating strategies, such as one way, zig-zag, and back-and-forth. One-way method was used in this study. In contrast to materials that require high heat flow, Inconel 718 and titanium alloys can be preheated with a one-way preheating method. It was set up to maintain a focal length of 138 mm in order to obtain a proper preheating effect.

Table 4 shows the preheating temperature and feed rate used for the thermal effect experiment. The control of the laser and induction heat sources was by the temperature control method, and the power was automatically maintained by a pyrometer. Temperature measurements were conducted with the laser or induction heat source moving along the center of the top surface on the workpiece. 

The laser-induction assisted milling using multi heat sources was classified into two methods according to the order of the heat source. In the first method the induction heat source was preceded by a laser (Method 1), and in the second the laser heat source was preceded by an induction (Method 2).

### 3.2. Results and Discussion

In this chapter, a thermal analysis model for proper DOC determination and verification of temperature distribution was constructed and this model is experimentally verified. In TAM, the surface area to be preheated is cut by the cutting tool. Also, a depth of 1 mm or more is not included in the cutting range and the heat effect is relatively small. As a result, the error at a depth of 0.5 mm was estimated.

#### 3.2.1. Inconel 718

**Figure 7:**
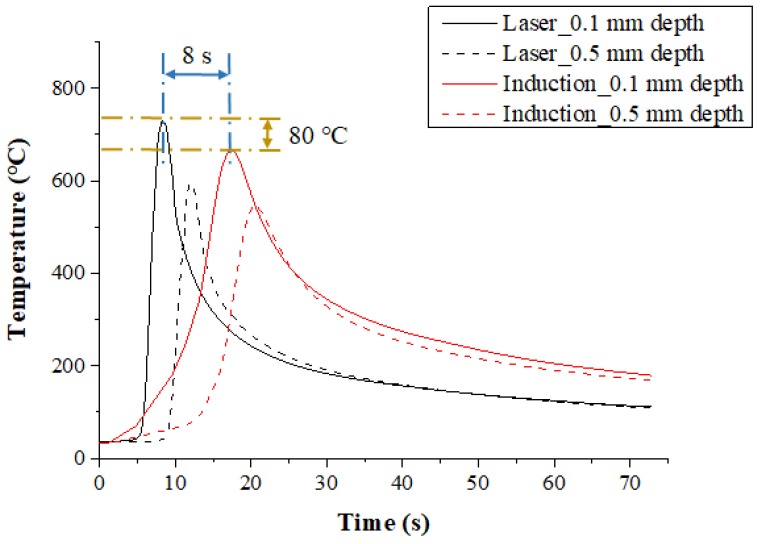
The temperature distribution for a single heat source (laser or Induction) in Inconel 718 (feed rate: 200 mm/min).

The temperature distribution experiments with Inconel 718 were performed with the various heat sources types. Figure 7 shows the measured temperature at depths of 0.1 mm and 0.5 mm from the surface of the Inconel 718 for the single preheating method, using a laser or induction. The feed rate was set at 200 mm/min. When the single heat source of laser and induction was used, the preheating effect and the temperature maintenance after the irradiation of the heat source were compared. For the laser, the maximum temperature was increased by approximately 80 °C compared to the induction heat source at the same feed rate, by concentrating the heat source on the workpiece. Also, in comparison with induction, it was not necessary to preheat the workpiece, so the heat source could be irradiated at the set temperature at the beginning of the machining.

For the induction, a preheating time of about 8 seconds more was required to reach the set temperature as compared with the laser, and the maximum temperature was lower than that of laser, as the heat source is more dispersed. However, after the workpiece was preheated, maintaining the temperature of the workpiece was better using induction. The temperature of the preheated workpiece was maintained for approximately twice as long as that of the laser. When the preheating temperature of the material is maintained longer, the heat source to be supplied is increased, so that high machining efficiency could be obtained for the difficult-to-cut materials.

**Figure 8:**
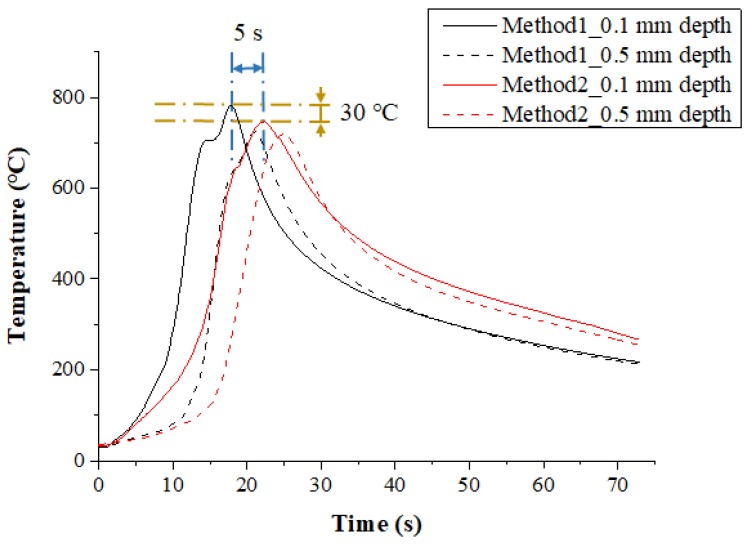
The temperature distribution of the multi heat sources according to preheating order for laser and induction in Inconel 718 (feed rate: 200 mm/min).

Figure 8 shows the measured temperature at a depth of 0.1 mm and 0.5 mm from the surface of the Inconel 718 following the multi preheating method of laser-induction. In order to optimize the advantages of each single heat source, a thermal effect analysis of multi heat sources using laser and induction was carried out. The preheating effect of Method 1, in which induction was used as an auxiliary heat source, was the most effective. Compared with Method 2, which used induction as the main heat source, the material preheating time was about 5 s shorter, and the preheating effect was slightly better as the feed rate increased. Also, the temperature was maintained better than with a single laser heat source.

**Figure 9:**
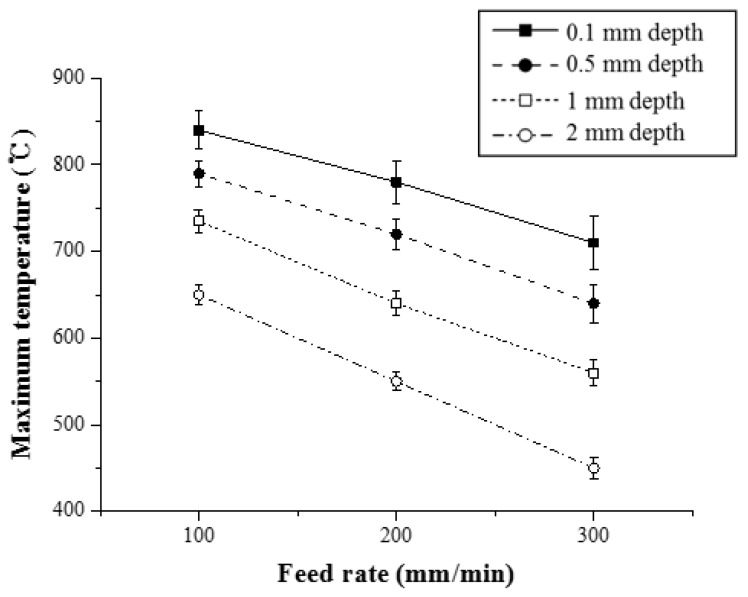
The maximum temperature with respect to the feed rate for Inconel 718 (Method 1).

In order to analyze the thermal effect of the multi heat sources, Figure 9 shows the maximum temperature with respect to the feed rate for Inconel 718 (Method 1). As the feed rate of the heat source increased, the preheating effect of the workpiece decreased. When the feed rate was increased from 100 to 200 mm/min and from 200 to 300 mm/min, the maximum temperature at a depth of 0.1 mm from the workpiece surface was reduced by 7% and 10.1%, respectively. At a depth of 0.5 mm, the maximum temperature was reduced by 9.8% and 11%, respectively. When the workpiece was irradiated with the same preheating temperature, the preheating effect was reduced because the heat input absorbed by the material decreased as the feed rate increased.

#### 3.2.2. Ti-6Al-4V

**Figure 10:**
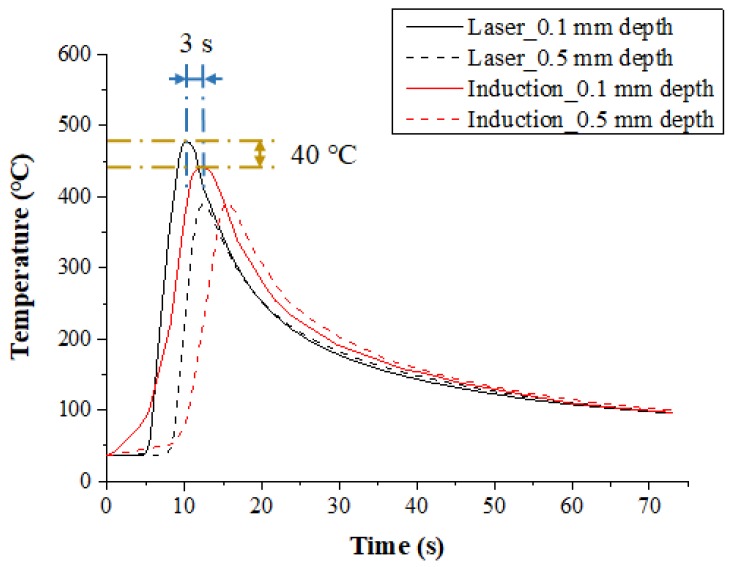
The temperature distribution for a single heat source (laser or Induction) in Ti-6Al-4V (feed rate: 200 mm/min).

The temperature distribution experiments with Ti-6Al-4V were performed according to heat sources types. Figure 10 shows the measured temperature at a depth of 0.1 mm and 0.5 mm from the surface of the Ti-6Al-4V according to the single preheating method of laser and induction. The feed rate was set at 200 mm/min. When comparing the two single heat sources, there was no significant difference in the preheating effect. For a laser, the maximum temperature was increased by approximately 40 °C compared to induction in the same feed rate. For an induction, the preheating time was required to be about 3 seconds more to reach the set temperature compared with the laser. Because the set temperature was not high, the preheating time of the workpiece at the beginning of machining was not long. Also, the time of temperature maintenance after induction heating of the workpiece was similar to that of the laser heat source. 

**Figure 11:**
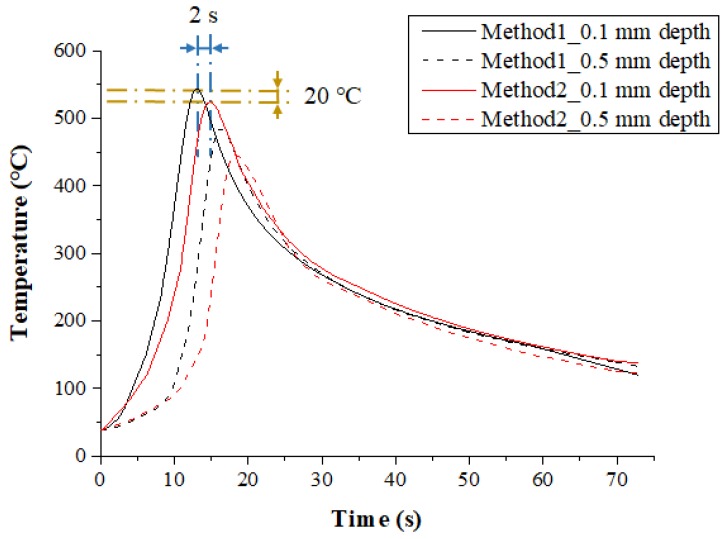
The temperature distribution of the multi heat sources according to preheating order for laser and induction in Ti-6Al-4V (feed rate: 200 mm/min).

Figure 11 shows the measured temperature at depths of 0.1 mm and 0.5 mm from the surface of Ti-6Al-4V according to the multi preheating methods of laser-induction. The preheating effect was similar for both methods because the set temperature was not high. However, the maximum temperature of Method 1 with laser as the main heat source was 20 °C and 40 °C higher than Method 2 at the depths of 0.1 mm and 0.5 mm from workpiece surface, respectively.

**Figure 12:**
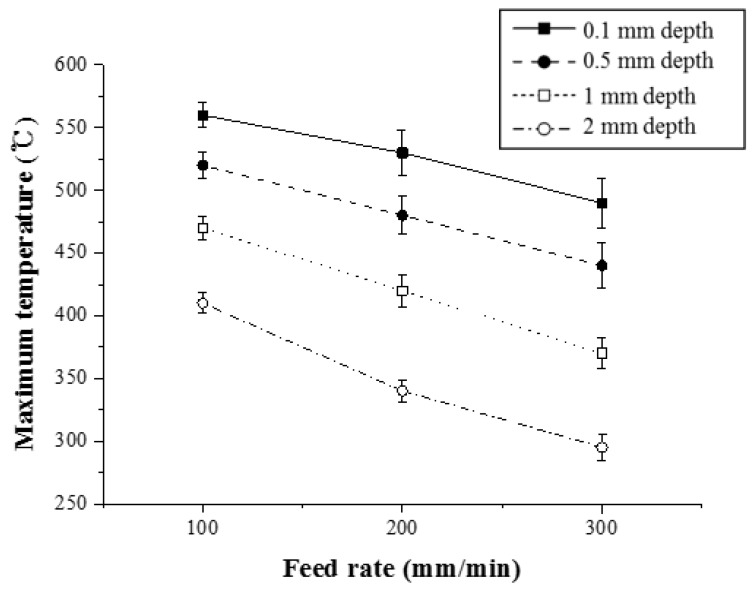
The maximum temperature with respect to the feed rate for Ti-6Al-4V (Method 1).

Figure 12 shows the maximum temperature with respect to the feed rate for Ti-6Al-4V (Method 1). For a depth of 0.5 mm, the maximum temperature was reduced by 8.4% and 10.2%, respectively.

### 3.3. Comparison of the Thermal Analysis and Measurements Experiment Results

The thermal analysis results obtained by FEM and the measured maximum temperature using thermocouples were compared. The effectiveness of the electromagnetic-thermal coupled analysis was verified by comparing the measured maximum temperature with the thermal analysis results. The feed rate of the multi heat sources using laser and induction was 200 mm/min. A comparison was made between the maximum temperature in the thermal analysis and the experimental values measured at depths of 0.1 mm, 0.5 mm, 1 mm, and 2 mm from the workpiece surface, respectively. In the thermal analysis, the nodes corresponding to the set depths were selected and the temperature distribution over time was measured. The comparison model between thermal analysis and the experiment is Method 1. This analysis was performed to verify the analytical model through experiments. Method 1 was chosen as a representative case. In addition, the surface area to be preheated is cut by the cutting tool. Also, a depth of 1 mm or more is not included in the cutting range and the heat effect is relatively small. As a result, the error at depth of 0.5mm was estimated to be less than 2.5% and it was verified as an appropriate analysis model.

#### 3.3.1. Inconel 718

**Figure 13:**
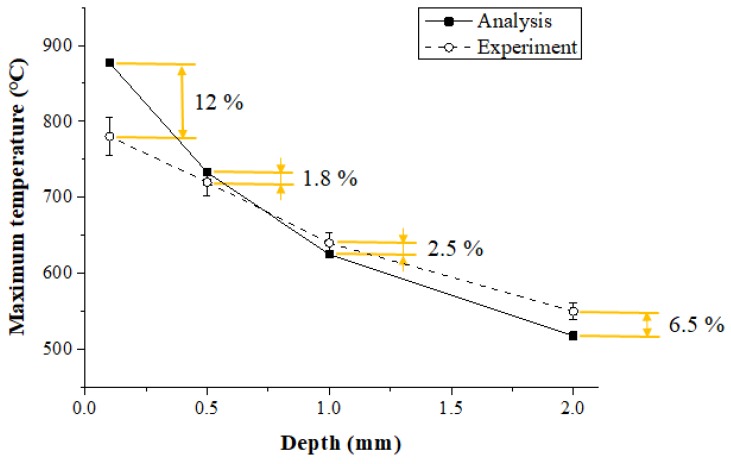
The maximum temperature of thermal analysis and experimental values according to measurement depth of Inconel 718 (feed rate: 200 mm/min).

Figure 13 shows the maximum temperature in the thermal analysis and experimental values according to the measurement depth of Inconel 718. When the depth of the workpiece was 0.1 mm, 0.5 mm, 1 mm, and 2 mm, the error between the thermal analysis and experimental values was 12%, 1.8%, 2.5%, and 6.5%, respectively. When the depth was between 0.5 and 1 mm, the error was within approximately 2.5%, and the analysis results were similar to the measured experimental values. The error in the results increased with the surface area of the workpiece and the error increased as the depth became deeper than 1 mm. The surface temperature set in the thermal analysis was 900 °C, but the preheating effect produced by actual temperature control was reduced as the feed rate increased.

#### 3.3.2. Ti-6Al-4V

**Figure 14:**
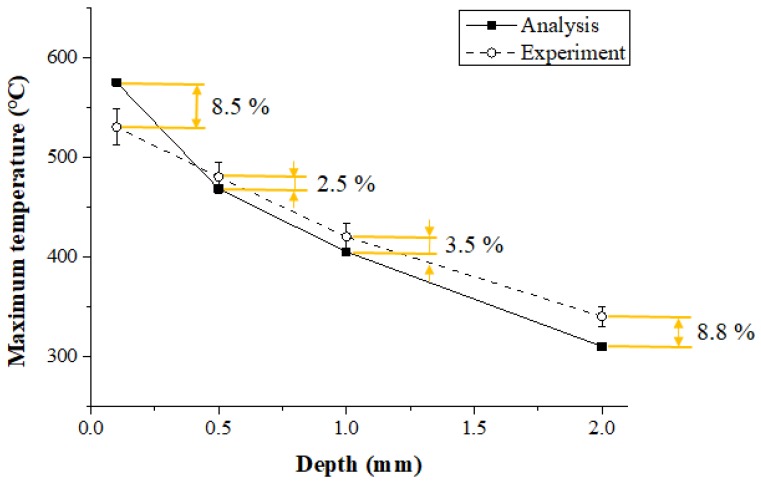
The maximum temperature of thermal analysis and experimental values according to measurement depth of Ti-6Al-4V (feed rate: 200 mm/min).

Figure 14 shows the maximum temperature of the thermal analysis and the experimental values according to measurement depth in the Ti-6Al-4V. When the depth of the workpiece was 0.1 mm, 0.5 mm, 1 mm, and 2 mm, the error between the thermal analysis and experimental values was 8.5%, 2.5%, 3.5%, and 8.8%, respectively. Within 1 mm of the actual cutting depth, the error between the thermal analysis and experiment results was within 3.5%, and the error increased as the depth became deeper than 1 mm.

## 4. Thermally Assisted Machining

### 4.1. Experimental Set-Up

**Figure 15:**
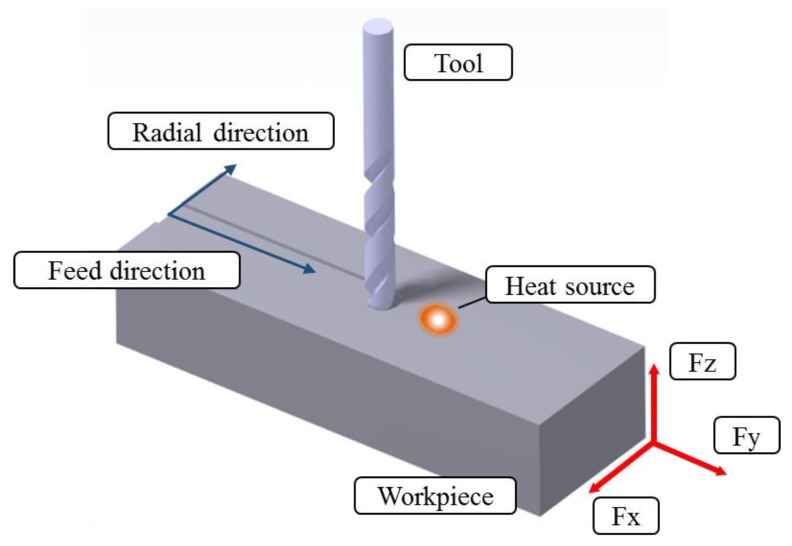
Coordinates of tool dynamometer.

TAM methods, such as LAM, induction assisted machining (IAM), and laser induction assisted machining (LIAM), were performed to compare the machining characteristics of the four types of heat sources. The objective of the TAM is to remove the desired machining area without changing the material properties of the machined workpieces after preheating locally. Therefore, analyzation of the heat affective zone through the thermal analysis and the preheating experiment was carried out before the TAM. During TAM, the area where changes in the material properties occurs, including phase transformation and the increase of residual stresses, was removed by the following cutting tool. The equipment for machining was the same model as that shown in Table 3. In the machining, the thermocouples used for the thermal measurement were not used, and instead a tool dynamometer (KISTLER Co., Ltd., Winterthur, Switzerland, 9257) for measuring the cutting force was used. It was installed on the index table of the 5-axis machining center. Also, Surface roughness equipment (OPTACOM GmbH & Co., Grettstadt, Germany, VC-10) and a microscope (ANMO ELECTRONICS Co., Ltd., New Taipei City, Taiwan, Dino-lite; AM4113T) were used to analyze the surface of the workpiece. The feed rate of the heat sources and the tool attached to the spindle, and the preheating temperature according to each heat source type were applied using the same conditions as those in Table 4. The spindle speed was 4000 rpm and the 8Ø end-mill (WIDIN Co., Ltd., Changwon, Korea, type ZE324081) was used. The cutting speed was applied at 101 m/min in all experimental conditions in order to analyze the machining conditions according to the change in feed rate. Figure 15 shows the feed direction and radial direction of the workpiece in which the tool dynamometer was installed.

The distance between the edge of the cutting tool and the heat source was determined to be 3 mm by FEM simulation and experiments for various kinds of cutting tool and materials designed to reduce the thermal effect on the cutting tool.

### 4.2. Inconel 718

#### 4.2.1. Cutting Forces

**Figure 16:**
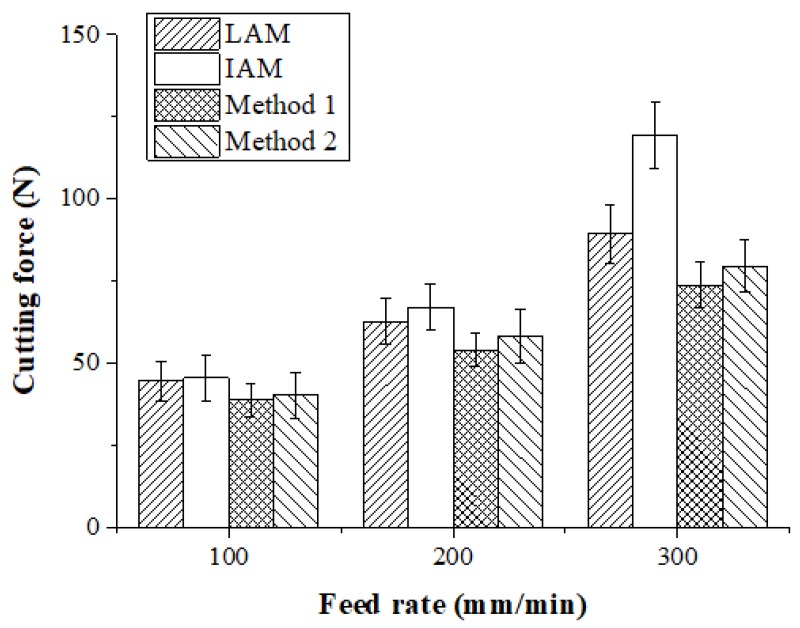
Cutting force of Inconel 718 according to the four types of heat sources.

The machining of Inconel 718 was carried out and the cutting forces were measured using a tool dynamometer. Figure 16 shows the measured cutting forces according to the four types of heat sources. The cutting force was selected as the resultant force of the feed direction cutting force (Fx) and the radial direction cutting force (Fy). Because the DOC was constant during machining, the average values of Fx and Fy were used. The cutting force according to the feed rate of the heat source was lowest in the laser-induction assisted milling, using induction as an auxiliary heat source. At a feed rate of 100 mm/min, the cutting force of LAM was 44.72 N, the cutting force of IAM was 62.65 N, the cutting force of Method 1 was 38.83 N, and the cutting force of Method 2 was 40.31 N. The cutting force of Method 1 was reduced by approximately 15%, 16%, and 4% compared to LAM, IAM, and Method 2, respectively. At a feed rate of 200 mm/min, the cutting force of LAM was 62.64 N, the cutting force of IAM was 67.08 N, the cutting force of Method 1 was 54.08 N, and the cutting force of Method 2 was 58.31 N. The cutting force of Method 1 was reduced by about 16%, 24%, and 8% compared to LAM, IAM, and Method 2, respectively. At a feed rate of 300 mm/min, cutting force of LAM was 89.44 N, cutting force of IAM was 67.08N, cutting force of Method 1 was 54.08 N and cutting force of Method 2 was 79.612 N. The cutting force was reduced by about 21%, 62%, and 8%, respectively.

As the feed rate increased, the preheating effect of the heat source decreased and the cutting force increased. In particular, due to the characteristics of the induction that dispersed the heat source, as the feed increased, the preheating effect sharply decreased, so the cutting force greatly increased compared to other heat sources. For multi heat sources, the cutting force was lower than the single heat source in both methods because the temperature of the workpiece was better maintained than with the single heat source. At a feed rate of 100 mm/min, the cutting force of a single laser or induction heat source was not significantly different, averaging about 5%. If the feed rate is low, a high preheating effect can be obtained by using an induction device, which is cheaper than a laser.

#### 4.2.2. Surface Roughness

**Figure 17:**
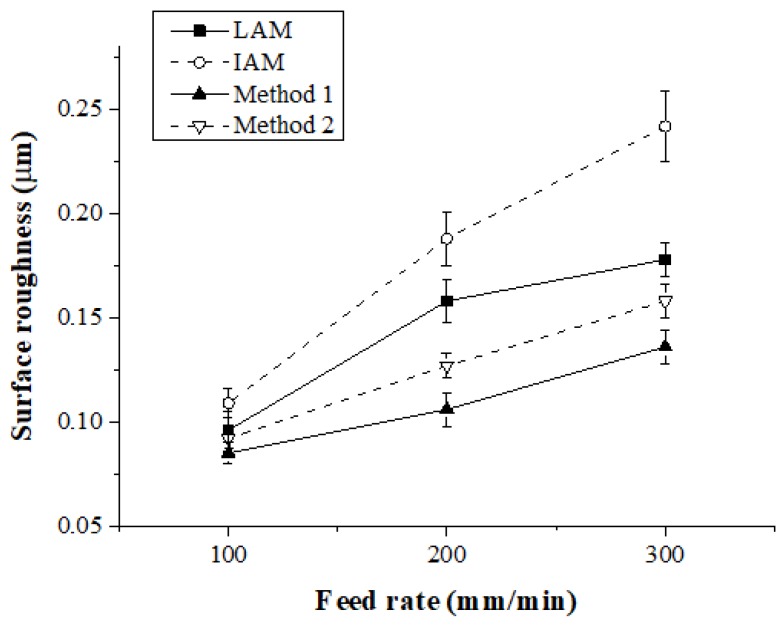
Surface roughness of Inconel 718 according to the four types of heat sources.

The surface roughness was measured by average height (Ra). The measurement length of the surface roughness was measured at 3 mm at the same position on the workpiece. Figure 17 and Table 5 show the surface roughness according to the feed rate for each heat source type. When the feed rate was 100 mm/min, the surface roughness was similarly measured to be approximately 0.1 μm for the four types of heat sources. When the feed rate was 200 mm/min, the surface roughness for Method 1 was improved compared to LAM, IAM, and Method 2 by approximately 33%, 43%, and 16%, respectively. At a feed rate of 300 mm/min, the surface roughness was improved about 23%, 44%, and 14%, respectively. The surface roughness was also improved with the multi heat sources compared with the single heat source, like the cutting force. With the multi heat sources, before the tool cuts a workpiece, the irradiated preheating value is greater than a single heat source. Therefore, the surface roughness is further improved because the cutting resistance is reduced. 

**Figure 18:**
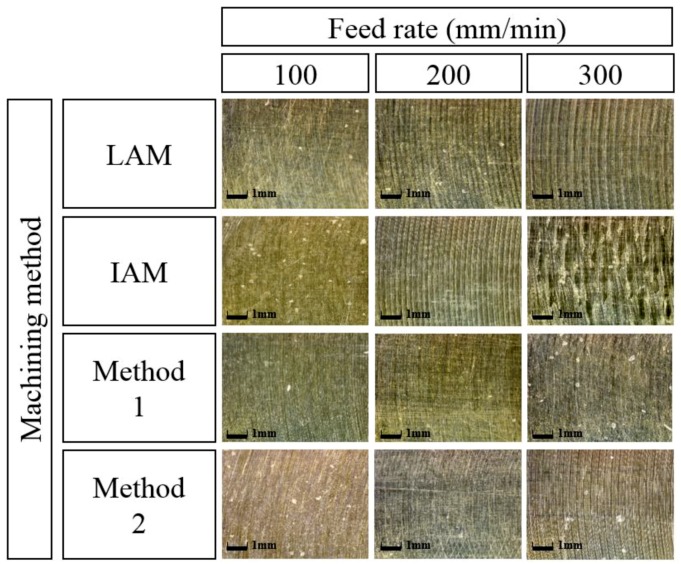
Microphotographs of machined surfaces of Inconel 718 according to feed rate.

Figure 18 shows a microphotograph of the machined surface according to the heat source types. The magnification scale of the microscope was set at 300 to measure the workpiece surface. As the feed rate increases, it can be seen that the machined surface of the multi heat sources is improved in comparison with a single heat source.

### 4.3. Ti-6Al-4V

#### 4.3.1. Cutting Forces

**Figure 19:**
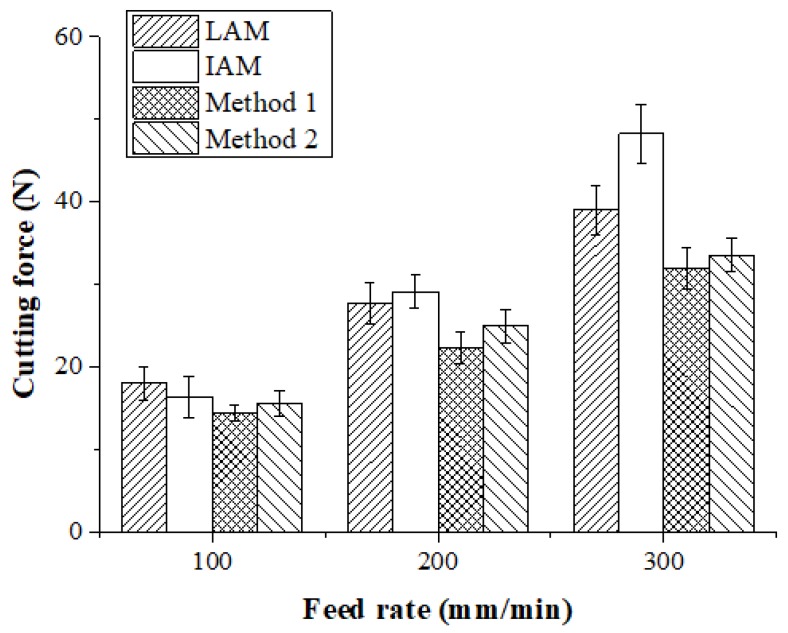
Cutting force of Ti-6Al-4V according to the four types of heat sources.

The machining of Ti-6Al-4V was carried out by TAM. Figure 19 and Table 6 show the measured cutting forces according to the four types of heat sources. At a feed rate of 100 mm/min, the cutting force of LAM was 18.03 N, the cutting force of IAM was 29.15 N, the cutting force of Method 1 was 22.36 N, and the cutting force of Method 2 was 15.62 N. The cutting force of Method 1 was decreased by approximately 25%, 14%, and 5% in comparison with LAM, IAM, and Method 2, respectively. At a feed rate of 200 mm/min, the cutting force of LAM was 27.80 N, the cutting force of IAM was 29.15 N, the cutting force of Method 1 was 22.36 N, and the cutting force of Method 2 was 25.00 N. The cutting force of Method 1 was reduced by about 24%, 30%, and 12% compared to LAM, IAM, and Method 2, respectively. At a feed rate of 300 mm/min, the cutting force of LAM was 39.05 N, the cutting force of IAM was 48.26N, the cutting force of Method 1 was 32.06 N, and the cutting force of Method 2 was 33.54 N. The cutting force of Method 1 was reduced by about 22%, 51%, and 5% compared to LAM, IAM, and Method 2, respectively. When the feed rate was 100 mm/min, the cutting force of the induction heat source was lower than that of the laser, indicating that induction can achieve a preheating effect similar to the laser when the feed is low. As the feed rate increases, the preheating effect of the multi heat sources increased more than the single heat source. 

#### 4.3.2. Surface Roughness

**Figure 20:**
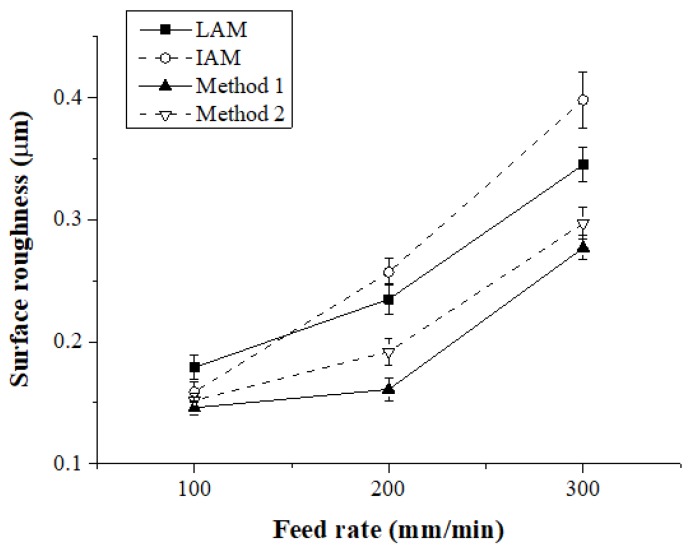
Surface roughness of Ti-6Al-4V according to the four types of heat sources.

Figure 20 shows the surface roughness according to the feed rate for each heat source type. When the feed rate was 100 mm/min, the surface roughness of IAM, Method 1, and Method 2 were similar, and the surface roughness of LAM was approximately 13% higher than other heat sources. At a feed rate of 200 mm/min, the surface roughness of Method 1 compared to LAM, IAM, and Method 2 was improved by about 31%, 37%, and 16%, respectively, and at a feed rate of 300 mm/min, the surface roughness was improved approximately 19%, 30%, and 6%, respectively.

**Figure 21:**
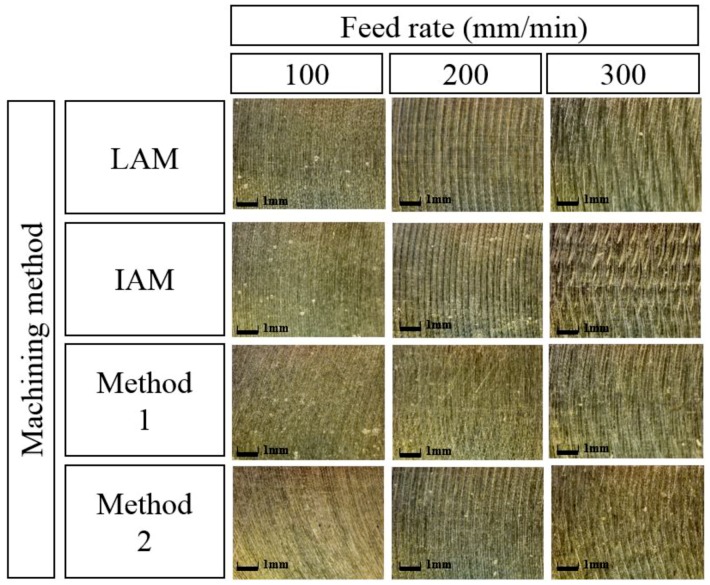
Microphotographs of machined surfaces of Ti-6Al-4V according to feed rate.

Figure 21 shows a microphotograph of the machined surface according to the types of heat sources. As shown in the figure, the machined surface of the laser-induction assisted milling, in which the induction was used as an auxiliary heat source, is seen to be the best result.

## 5. Conclusions

In this study, the thermal effect analysis of single heat source and multi heat sources using laser and induction was performed, and a machining experiment was carried out to analyze the machining characteristics of Inconel 718 and Ti-6Al-4V materials according to heat source types. The thermal analysis was performed to select the proper preheating temperature and effective depth of cut before the machining experiment. Experiments were performed by varying the feed rate of the heat sources, and the machining characteristics, such as cutting force and surface roughness, were analyzed from the measurement results. The conclusions obtained from this study are as follows.

(1) A proper preheating temperature and effective depth of cut were obtained by FEM, considering the tensile strength of Inconel 718 and Ti-6Al-4V materials, and the thermal analysis results were applied to the machining experiments.

(2) The workpiece was not intensively irradiated. As such a longer preheating time was needed to heat the workpiece, and the preheating effect decreased sharply as the feed rate was increased. For multi heat sources with combined laser and induction, the total heat flow of the heat source irradiated to the workpiece was higher than a single heat source. Therefore, when multi heat sources are used, rapid temperature reduction can be prevented and TAM efficiency can be increased.

(3) The effectiveness of the thermal analysis was verified by comparing the maximum temperatures of the thermal analysis and experiment results. When the depth was between 0.5 and 1 mm, the error was within approximately 3.5 %, and the thermal analysis results were similar to the measured experimental values.

(4) For Inconel 718, the cutting forces using Method 1 were reduced by up to 18%, 38%, and 7% compared to LAM, IAM, and Method 2, respectively, and the surface roughness was improved by up to 33%, 44%, and 16%, respectively. For Ti-6Al-4V, the cutting forces using Method 1 decreased by up to 20%, 34%, and 10% compared to LAM, IAM, and Method 2, respectively, and the surface roughness was improved by up to 31%, 37%, and 16%, respectively.

(5) Method 1, which used induction as an auxiliary heat source, had lower cutting forces and improved surface roughness compared to Method 2. At a feed rate of 100 mm/min, the cutting forces and surface roughness were similar regardless of heat source types. However, at a feed rate of 300 mm/min, the order of excellent machining characteristics was Method 1, Method 2, LAM, and IAM. This is because with induction, the preheating effect is drastically decreased as the feed rate is increased.

Inconel 718 requires a high preheating temperature requirement, so a better preheating effect and better surface roughness are obtained in multi heat sources than a single heat source. The preheating effect of multi heat source was better than that of single heat source.

Ti-6Al-4V requires a low preheating temperature compared to Inconel 718, so there are few significant differences between multi heat sources and single heat sources. Cutting force and surface roughness were effective using multi heat sources, but the difference in the preheating effect between the multiple heat source and the single heat source was relatively small.

Therefore, it is important to determine a proper preheating method, since the preheating characteristics differ depending on the thermal properties and the preheating temperature of the material.

The results of this study provide basic data for TAM for high-speed machining.

## Figures and Tables

**Table 1:** The specific heat and thermal conductivity of Inconel 718 [15].

Temperature (°C)	Thermal Conductivity (W/mK)	Specific Heat (J/kgK)
20	11.4	427.14
100	12.5	441.74
300	14	481.74
500	15.5	521.74
700	21.5	561.74
900	26.2	601.74
1350	31.3	691.74

**Table 2:** The specific heat and thermal conductivity of Ti-6Al-4V [28].

Temperature (°C)	Thermal Conductivity (W/mK)	Specific Heat (J/kgK)
25	7	546
100	7.45	562
300	10.15	606
500	12.6	651
700	15.5	694
900	20.2	734
1300	23.7	696
1500	25.8	732
1600	27	750

**Table 3:** Experimental equipment.

Equipment (Model)	Maker (Specifications)
5-axis machining center (Hi-V560M)	Hyundai-WIA
Laser module (LDM-1000-100)	Laserline (1kW diode laser, Wavelength: 940–980 nm)
Induction module (TH-6000)	Tae Yang induction (Frequency: 300 kHz)
Pyrometer (LPC03)	Dr. Mergenthaler GmbH & Co. KG (Temperature range: 400–3000 °C)
Thermocouples/Data logger (GL-220)	Graphtec (−100–1370 °C)

**Table 4:** Experimental conditions.

Material	Inconel 718, Ti-6Al-4V	
**Feed rate for laser and induction (mm/min)**	100, 200, 300	
**Preheating method**	**Preheating temperature of Inconel 718**	
	**Laser (°C)**	**Induction (°C)**
**Laser (only)**	900	-
**Induction (only)**	-	900
**Method 1**	900	700
**Method 2**	700	900
**Preheating method**	**Preheating temperature of Ti-6Al-4V**	
	**Laser (°C)**	**Induction (°C)**
**Laser (only)**	600	-
**Induction (only)**	-	600
**Method 1**	600	450
**Method 2**	450	600

**Table 5:** Surface roughness of Inconel 718.

	Machining Method			
Feed Rate (mm/min)	LAM	IAM	Method 1	Method 2
100	0.096	0.109	0.085	0.092
200	0.158	0.188	0.106	0.127
300	0.178	0.242	0.136	0.158

**Table 6:** Surface roughness of Ti-6Al-4V.

	Machining Method			
Feed Rate (mm/min)	LAM	IAM	Method 1	Method 2
100	0.096	0.109	0.085	0.092
200	0.158	0.188	0.106	0.127
300	0.178	0.242	0.136	0.158

## References

[1] Lee H., Lim C.H.J., Low M.J., Tham N., Murukeshan V.M., Kim Y.-J. (2017). Lasers in additive manufacturing: A review. Int. J. Precis. Eng. Manuf.-Gr. Technol..

[2] Pattanayak S., Panda S. (2018). Laser Beam Micro Drilling–A Review. Lasers Manuf. Mater. Process..

[3] Shelton J.A., Shin Y.C. (2010). Comparative evaluation of laser-assisted micro-milling for AISI 316, AISI 422, TI-6AL-4V and Inconel 718 in a side-cutting configuration. J. Micromech. Microeng..

[4] Xiao H., Li S., Han X., Mazumder J., Song L. (2017). Laves phase control of Inconel 718 alloy using quasi-continuous-wave laser additive manufacturing. Mater. Des..

[5] Guimaraes A.A., Jonas J.J. (1981). Recrystallization and aging effects associated with the high temperature deformation of Waspaloy and Inconel 718. Metall. Trans. A..

[6] Ayed Y., Germain G., Ben Salem W., Hamdi H. (2014). Experimental and numerical study of laser-assisted machining of Ti6Al4V titanium alloy. Finite Elem. Anal. Des..

[7] Lee S.J., Kim J.D., Suh J. (2014). Microstructural variations and machining characteristics of silicon nitride ceramics from increasing the temperature in laser assisted machining. Int. J. Precis. Eng. Manuf..

[8] Venkatesan K., Ramanujam R., Kuppan P. (2016). Parametric modeling and optimization of laser scanning parameters during laser assisted machining of Inconel 718. Opt. Laser Technol..

[9] Tadavani S.A., Razavi R.S., Vafaei R. (2017). Pulsed laser-assisted machining of Inconel 718 super alloy. Opt. Laser Technol..

[10] Bermingham M.J., Schaffarzyk P., Palanisamy S., Dargusch M.S. (2014). Laser-assisted milling strategies with different cutting tool paths. Int. J. Adv. Manuf. Technol..

[11] Kim I.W., Lee C.M. (2017). Investigation into the machining characteristics of AISI 1045 steel and Inconel 718 for an ellipsoidal shape using laser-assisted contouring and ramping machining. Int. J. Precis. Eng. Manuf..

[12] Woo W.S., Lee C.M. (2015). A study of the machining characteristics of AISI 1045 steel and Inconel 718 with a cylindrical shape in laser-assisted milling. Appl. Therm. Eng..

[13] Wiedenmann R., Zaeh M.F. (2015). Laser-assisted milling-Process modeling and experimental validation. CIRP J. Manuf. Sci. Technol..

[14] Kong X., Yang L., Zhang H., Zhou K., Wang Y. (2015). Cutting performance and coated tool wear mechanisms in laser-assisted milling K24 nickel-based superalloy. Int. J. Adv. Manuf. Technol..

[15] Kim D.H., Lee C.M. (2014). A study of cutting force and preheating-temperature prediction for laser-assisted milling of Inconel 718 and AISI 1045 steel. Int. J. Heat Mass Transf..

[16] Park K.H., Yang G.D., Lee M.G., Jeong H., Lee S.W., Lee D.Y. (2014). Eco-friendly face milling of titanium alloy. Int. J. Precis. Eng. Manuf..

[17] Ding H., Shen N., Shin Y.C. (2012). Thermal and mechanical modeling analysis of laser-assisted micro-milling of difficult-to-machine alloys. J. Mater. Process. Technol..

[18] Lee C.M., Kim D.H., Baek J.T., Kim E.J. (2016). Laser assisted milling device: A review. Int. J. Precis. Eng. Manuf.-Gr. Technol..

[19] Bermingham M.J., Sim W.M., Kent D., Gardiner S., Dargusch M.S. (2015). Tool life and wear mechanisms in laser assisted milling Ti–6Al–4V. Wear.

[20] Research report of the National Research Foundation of Korea (NRF) (2016). A New Conceptual 3-Dimensional Laser Assisted Mach. Syst..

[21] Kim D.H., Lee C.M. (2016). A study on the laser-assisted ball-end milling of difficult-to-cut materials using a new back-and-forth preheating method. Int. J. Adv. Manuf. Technol..

[22] Hwang S.J., Oh W.J., Lee C.M. (2016). A study of preheating characteristics according to various preheating methods for laser-assisted machining. Int. J. Adv. Manuf. Technol..

[23] Loópez de Lacalle L.N., Saánchez J.A., Lamikiz A., Celaya A. (2004). Plasma assisted milling of heat-resistant superalloys. Trans. ASME.

[24] Ginta T.L., Amin A.K.M.N. (2013). Thermally-assisted end milling of titanium alloy Ti-6Al-4V using induction heating. Int. J. Mach. Mach. Mater..

[25] Kim E.J., Lee C.M. (2019). A Study on the Optimal Machining Parameters of the Induction Assisted Milling with Inconel 718. Materials.

[26] López de Lacalle L.N., Lamikiz A., Celaya A. (2002). Simulation of Plasma Assisted Milling of Heat Resistnat Alloys. Int. J. Simul. Modell..

[27] Hedberg G.K., Shin Y.C. (2015). Laser assisted milling of Ti-6Al-4V ELI with the analysis of surface integrity and its economics. Lasers Manuf. Mater. Process..

[28] Yang J., Sun S., Brandt M., Yan W. (2010). Experimental investigation and 3D finite element prediction of the heat affected zone during laser assisted machining of Ti6Al4V alloy. J. Mater. Process. Technol..

[29] Oh H.S., Cho H.R., Park H., Hong S.T., Chun D.M. (2016). Study of electrically-assisted indentation for surface texturing. Int. J. Precis. Eng. Manuf.-Gr. Technol..

[30] Shen N., Ding H. (2013). Thermo-mechanical coupled analysis of laser-assisted mechanical micromilling of difficult-to-machine metal alloys used for bio-implant. Int. J. Precis. Eng. Manuf..

[31] Kang D.W., Lee C.M. (2013). Study on determining the exponents for a constitutive equation in laser assisted machining. Int. J. Precis. Eng. Manuf..

[32] Calleja A., Tabernero I., Ealo J.A., Campa F.J., Lamikiz A., López de Lacalle L.N. (2014). Feed rate calculation algorithm for the homogeneous material deposition of blisk blades by 5-axis laser cladding. Int. J. Adv. Manuf. Tech..

[33] Yoder G.R., Cooley L.A., Crooker T.W. (1977). Observations on microstructurally sensitive fatigue crack growth in a widmanstätten Ti-6Al-4V alloy. Metall. Trans. A.

[34] Zaeh M.F., Wiedenmann R., Daub R. (2010). A thermal simulation model for laser-assisted milling. Phys. Procedia.

[35] Tagliaferri F., Leopardi G., Semmler U., Kuhl M., Palumbo B. (2013). Study of the influences of laser parameters on laser assisted machining processes. Proced. CIRP.

